# Suitability of stability assessment methods for topical formulations enriched with apple pomace extract

**DOI:** 10.1371/journal.pone.0351678

**Published:** 2026-06-24

**Authors:** Katarzyna Czerniewicz, Anna Olejnik, Maria Urbańska, Karolina Latanowicz, Justyna Gornowicz-Porowska, Krzysztof Kus

**Affiliations:** 1 Department and Division of Pharmacoeconomics and Social Pharmacy, Poznan University of Medical Sciences, Poznań, Poland; 2 Department and Division of Practical Cosmetology and Skin Diseases Prophylaxis, Poznan University of Medical Sciences, Poznan, Poland; 3 Faculty of Chemistry, Adam Mickiewicz University in Poznan, Uniwersytetu Poznanskiego 8, Poznan, Poland; 4 Centre for Advanced Technologies, Adam Mickiewicz University in Poznan, Uniwersytetu Poznanskiego 8, Poznan, Poland; 5 Latech Company, Warszawa, Poland; National University of Rosario, ARGENTINA

## Abstract

The cosmetic industry has shown a growing interest in incorporating natural ingredients derived from food waste due to their perceived benefits and consumer preference for eco-friendly and sustainable products. Apple pomace, a by-product of apple juice and cider production, is rich in bioactive compounds such as polyphenols, mainly flavonoids and triterpenoids, offering antioxidant, anti-aging and anti-inflammatory benefits. The study aimed to develop and compare the stability of topical formulations containing apple pomace extract, including a cleansing gel, a serum, and a face cream (1%, 3% and 2% w/w extract, respectively), formulated in stable (A) and unstable (B) variants and to assess the effectiveness and efficiency of advanced stability testing methods for cosmetics. The key distinction in cleansing gels was the use of either Guar Hydroxypropyltrimonium Chloride or Cyamopsis Gum Tetragonoloba; in serums, stability depended on xanthan gum or Cyamopsis Gum Tetragonoloba; and for face creams, the critical factor was the incorporation of Cetearyl Olivate and Sorbitan Olivate. Formulations were stored for 30 days at 4°C, 25°C, and 45°C and analyzed using multiple light scattering (MLS), laser diffraction (LD) and optical microscopy. MLS confirmed high stability of all A variants, with negligible fluctuations in their transmission/backscattering profiles, whereas B variants exhibited pronounced instability reflected by marked changes in transmission signals (up to 58.6%). Optical microscopy confirmed these findings, with homogeneous droplet distribution in Cream A and heterogeneous coalescence in Cream B. These results demonstrate that MLS and LD were suitable for detecting changes in both emulsions and hydrogels, whereas optical microscopy was effective only for emulsions. The application of these advanced stability measurement techniques can enhance cosmetic formulation design and support sustainable cosmetic development.

## 1. Introduction

In recent years, the interest in utilizing fruit waste has increased significantly, driven by both environmental concerns and the potential to discover novel bioactive compounds [1,2]. Apple pomace (AP), the by-product of apple juice and cider production, is one such waste material that has garnered attention due to its rich content of active substances [3]. Traditionally used as animal feed, apple pomace is repurposed in an innovative way as a valuable ingredient for cosmetic formulations, showcasing its potential in less conventional applications [4]. Bioactive compounds derived from apple pomace are known for their beneficial properties in skincare, including anti-aging, moisturizing, and anti-inflammatory effects [5,6]. The growing trend towards sustainability and the use of eco-friendly ingredients in cosmetics further underscores the importance of finding viable applications for such waste products [7].

Apple pomace contains a variety of active substances that contribute to its efficacy as a cosmetic ingredient [8]. Polyphenols, for example, are well-known for their antioxidant properties, which help to neutralize free radicals and protect the skin from oxidative stress [9–12]. Flavonoids possess anti-inflammatory properties, which can soothe irritated skin and reduce redness [13–15]. Additionally, the high content of natural sugars and organic acids in apple pomace can enhance the moisturizing properties of cosmetic formulations. Interestingly, as was confirmed by *in vitro* study on human skin fibroblasts, apple pomace induces the proliferation of fibroblasts and production of factors related to skin anti-aging (type I collagen and hyaluronan) [16]. These characteristics make apple pomace an attractive ingredient for the development of natural and effective skincare products [17–19]. The literature shows that AP has been explored mainly in the food sector (as a fiber- and polyphenol-rich ingredient, and for product fortification) [15], including yogurts [20,21], short-dough biscuits and functional bread [22,23] with only limited, mostly proof-of-concept work on cosmetic uses. In several of these applications, apple pomace also acts as a structuring or clean-label stabilizing agent, improving texture, water retention and rheology, or forming nanofibrillar Pickering stabilizers [24–26]. In cosmetics-relevant studies, apple pomace has been processed into powders/particles to improve water retention and rheology—positioning it as a natural cosmetic ingredient [26]—and AP extract has shown skin-relevant bioactivity *in vitro* (human dermal fibroblasts; increased proliferation and ECM markers) [27]. These reports indicate feasibility but remain single-product or exploratory. To our knowledge, no prior study has developed an integrated series of mutually compatible cosmetic products (a routine) built on a standardized AP extract and supported by coordinated, market-ready stability data suitable for near-term launch.

The cosmetic industry, responding to the growing consumer preference for natural and eco-friendly products, is increasingly exploring the incorporation of such natural ingredients. Consumers today are more informed and conscientious about the ingredients in their skincare products, often favouring those that are derived from sustainable sources. This shift in consumer behaviour is pushing cosmetic manufacturers to innovate and incorporate plant-derived components into their formulations [28–30]. However, the successful application of such ingredients depends on the stability of formulations, which is critical for ensuring product efficacy, safety, and shelf-life. Natural active substances are particularly vulnerable to degradation from factors like light, heat, and oxygen, making thorough stability testing essential to preserve their beneficial properties [31,32].

A stable emulsion shows no significant changes in the system. It has a uniform distribution of the dispersed phase within the continuous phase. Stability is ensured by the appropriate selection of ingredients. Additionally, the stability of the emulsion is also influenced by the size of its particles—generally, the smaller the particles, the greater the expected stability. Over time, emulsions may undergo destabilization processes. Some of these processes, such as creaming, sedimentation, and flocculation, are reversible. This means that after proper mixing or homogenization, the system can be restored to its previous state. Irreversible changes include phase inversion, emulsion separation, and the phenomenon of coalescence, which is the increase in droplet size due to aggregation. These instabilities cannot be reversed by mixing or homogenization [33].

The stability of cosmetic formulations can be assessed using a variety of techniques, each varying in complexity and precision. Traditional methods commonly employed in the cosmetic industry include centrifugation and thermal stress tests. Centrifugation exposes the formulation to centrifugal forces, accelerating phase separation in unstable emulsions, which can be visually assessed. This method is often combined with temperature control for more accurate results. Thermal tests, where samples are stored at various temperatures over extended periods, offer another way to observe changes in formulation stability. However, these conventional methods, while effective, require long observation periods and primarily rely on visual assessments. In recent years, more advanced instrumental techniques have been developed to expedite stability testing and provide more detailed insights into formulation behaviour. Multiple light scattering (MLS) and laser diffraction (LD) represent state-of-the-art methods that allow for real-time monitoring of particle size distribution and the detection of destabilization phenomena such as flocculation, coalescence, creaming, and sedimentation [34]. These techniques enable a comprehensive analysis in significantly less time than traditional approaches, often reducing the testing period to even within two weeks. Another advanced method is laser diffraction, that operates based on the principle of laser light diffraction, where the scattered light from particles in the sample is measured to determine their size distribution. This technique provides precise and reliable data on particle size, allowing for a detailed analysis of the formulation’s stability [35,36].

This study aimed to develop a series of cosmetic formulations containing apple pomace extract derived from the food industry, including hydrogels (cleansing gels, serums) and oil-water-emulsions (face creams), in both stable and unstable variants. The research focused on demonstrating how altering or removing a single formulation component can significantly influence the overall stability of the product. Advanced stability testing methods—multiple light scattering and laser diffraction—were employed to detect and evaluate these early changes. The study also aimed to highlight the potential of these modern techniques to optimize formulation processes and support the development of high-quality, sustainable cosmetic products for the cosmetic market.

## 2. Materials and methods

### 2.1. Materials

Studies were carried out on commercial apple pomace extract (Pomarage) kindly provided by Provital (Poznan, Poland). For the cosmetic formulations, the following ingredients were used: Euxyl K712, Euxyl PE9010, Euxyl K903, and N-Hance 3196 (Barentz, Hoofddorp, Holland); Sodium gluconate, Citric acid, Glycerine, and xanthan gum (Brenntag, Essen, Germany); Coco Glucoside AC818 and Olivem 1000 (HSH, Hamburg, Germany). Cetyl alcohol and Shea butter (Zarzeccy, Sieradz, Poland). Leraamph CAB 35 and Apriglu CG-GO 400v2 (Stockmeier, Neuss, Germany). Grape seed oil (Aurum Chemicals, Katowice, Poland), Jolee 775 (Surfachem, Leeds, UK), Panthenol 75 (Enzym, Koszalin, Poland), Hialuron DHA/BA 3 (Mabelle, Vitória, Brasil), Tegin M Pellets (Adara, Warsaw, Poland) and Fragrance (Arcon, Warsaw, Poland). All substances were stored according to manufacturers’ specifications until the formulating process.

### 2.2. Preparation of topical formulations

The study was conducted on topical facial formulations containing Pomarage, an apple pomace extract (INCI: Propanediol, Aqua, Apple Pomace Extract) obtained from apple pomace generated after apple pressing for cider production. The extract has a dark yellow colour and is water-soluble. It was incorporated into six formulations: two cleansing gels, two serums and two face creams, at 1%, 3% and 2% w/w, respectively, without additional pre-processing by the authors (Tables 1,2,3). Additionally, reference topical formulations were prepared without apple pomace extract using the same procedure (see Supporting Information). The chemical composition and quantity of each ingredient of these formulations are provided in the Supporting Information (Tables S1-S3 in S1 File).

**Table 1:** Chemical compositions of the cleansing gels.

No	Trade name	INCI	Company	Cleansing Gel A Quantity (% w/w, ± 0.01)	Cleansing Gel B Quantity (% w/w, ± 0.01)
1	Water	*Aqua*	–	80.09	80.09
2	Sodium gluconate	*Sodium Gluconate*	Brenntag	0.20	0.20
3	Glycerine	*Glycerine*	Brenntag	5.00	5.00
4	N-Hance 3196	*Guar Hydroxypropyltrimonium Chloride*	Barentz	1.30	–
5	Guar gum	*Cyamopsis Gum Tetragonoloba*	Barentz	–	1.30
6	Leraamph CAB 35	*Aqua, Cocamidopropyl Betaine, Sodium Chloride*	Stockmeier	3.00	3.00
7	Coco glucoside AC818	*Aqua, Coco Glucoside*	HSH	7.00	7.00
8	Apriglu CG-GO 400v2	*Aqua, Coco-Glucoside, Glyceryl Oleate, Citric Acid*	Stockmeier	0.50	0.50
9	Fragrance	*Parfum*	Arcon	0.20	0.20
10	Euxyl K712	*Sodium Benzoate, Potassium Sorbate, Aqua*	Barentz	1.50	1.50
11	Pomarage	*Propanediol, Aqua, Apple Pomace Extract*	Provital	1.00	1.00
12	Citric acid	*Citric Acid*	Brenntag	0.21	0.21

**Table 2:** Chemical compositions of the serums.

No	Trade name	INCI	Company	Serum A Quantity (% w/w, ± 0.01)	Serum B Quantity (% w/w, ± 0.01)
1	Water	*Aqua*	–	90.70	90.70
2	Sodium gluconate	*Sodium Gluconate*	Brenntag	0.20	0.20
3	Euxyl K712	*Sodium Benzoate, Potassium Sorbate, Aqua*	Barentz	0.50	0.50
4	Euxyl PE9010	*Phenoxyethanol, Ethylhexylglycerin*	Barentz	0.75	0.75
5	Glycerine	*Glycerine*	Brenntag	2.00	2.00
6	Xanthan gum	*Xanthan gum*	Brenntag	0.70	*–*
7	Guar gum	*Cyamopsis Gum Tetragonoloba*	Brenntag	–	0.70
8	Panthenol 75	*Panthenol*	Enzym	2.00	2.00
9	Pomarage	*Propanediol, Aqua, Apple Pomace Extract*	Provital	3.00	3.00
10	Fragrance	*Parfum*	Arcon	0.10	0.10
11	Citric acid	*Citric acid*	Brenntag	0.05	0.05

**Table 3:** Chemical compositions of the face creams.

No	Trade name	INCI	Company	Cream A Quantity (% w/w, ± 0.01)	Cream B Quantity (% w/w, ± 0.01)
1	Water	*Aqua*	–	73.70	77.70
2	Sodium gluconate	*Sodium Gluconate*	Brenntag	0.20	0.20
3	Euxyl K712	*Sodium Benzoate, Potassium Sorbate, Aqua*	Barentz	0.70	0.70
4	Euxyl K903	*Benzyl Alcohol, Benzoic Acid, Dehydroacetic Acid, Tocopherol*	Barentz	0.50	0.50
5	Olivem 1000	*Cetearyl Olivate, Sorbitan Olivate*	HSH	4.00	*–*
6	Tegin M Pellets	*Glyceryl Stearate*	Adara	1.00	1.00
7	Cetyl alcohol	*Cetyl alcohol*	Zarzeccy	2.00	2.00
8	Shea butter	*Butyrospermum Parkii Butter*	Zarzeccy	2.00	2.00
9	Grape seed oil	*Vitis Vinifera Seed Oil*	Aurum Chemicals	6.00	6.00
10	Jolee 7750	*Isoamyl Laurate*	Surfachem	4.00	4.00
11	Panthenol 75	*Panthenol*	Enzym	3.00	3.00
12	Hialuron DHA/BA 3	*Aqua, Sodium Hyaluronate, Hyaluronic Acid, Dehydroacetic Acid, Benzyl Alcohol*	Mabelle)	0.50	0.50
13	Pomarage	*Propanediol, Aqua, Apple Pomace Extract*	Provital	2.00	2.00
14	Fragrance	*Parfum*	Arcon	0.20	0.20
15	Citric acid	*Citric Acid*	Brenntag	0.20	0.20

#### 2.2.1. Cleansing gel A.

Sodium gluconate (2) was dissolved in distilled water (1), then glycerine (3) and N-Hance 3196 (4) were added. As a next step, the ingredients 6−11 were added. Afterwards, citric acid (12) was introduced until a pH of 5.0–5.5 was reached. The formulation was stirred with an automatic stirrer continuously throughout the whole procedure.

#### 2.2.2. Cleansing gel B.

Sodium gluconate (2) was dissolved in distilled water (1), then glycerine (3) and guar gum (5) were added. As a next step, the ingredients 6–11 were added. Afterwards, citric acid (12) was introduced until a pH between 5.0–5.5 was reached. The formulation was stirred with an automatic stirrer continuously throughout the whole procedure.

#### 2.2.3. Serum A.

Ingredients (2−6 and 8−10) were dissolved in distilled water (1) using an automatic stirrer. Afterwards, citric acid (11) was introduced until a pH between 5.0–5.5 was reached.

#### 2.2.4. Serum B.

Ingredients (2−5 and 7−10) were added under automatic stirrer to distilled water (1), then citric acid (11) was introduced until a pH between 5.0–5.5 was reached and stirred until the hydrogel formulation was obtained.

#### 2.2.5. Face cream A.

Distilled water (1) was heated to 70°C and sodium gluconate (2), Euxyl K712 (3), and Euxyl K903 (4) were added. At the same time, the ingredients of the oil phase (5−10) were heated to 70°C. When all the oil phase ingredients were melted, the water phase was added slowly and homogenized. Then the emulsion was cooled below 40°C and ingredients (11−14) were added, and the emulsion was homogenized. Afterwards, citric acid (15) was introduced until a pH between 5.0–5.5 was reached and the formulation was homogenized until the proper viscosity was fully developed.

#### 2.2.6. Face cream B.

Distilled water (1) and the oil phase (6−10) were heated to 70°C separately. To the water phase sodium gluconate (2), Euxyl K712 (3), and Euxyl K903 (4) were added. When all the oil phase ingredients were melted, the water phase was added slowly and homogenized. Then the emulsion was cooled below 40°C and ingredients (11−14) were added, and the emulsion was homogenized. Afterwards, citric acid (15) was introduced until a pH between 5.0–5.5 was reached and the formulation was homogenized until the proper viscosity was fully developed.

The samples, between measurements, were stored for a month (30 days) at different temperature regimes (4°C, 25°C, and 45°C) to conduct the stability studies.

### 2.3. Characterisation of topical formulations with apple pomace extract

#### 2.3.1. pH measurements.

The pH value of the formulations was measured at room temperature (RT) using a pH-meter (Elmetron CP-105, Poland). Each measurement was conducted in triplicate, and the average value was subsequently calculated and presented in Table 4.

**Table 4:** The pH values and refractive index of topical formulations.

Formulation	pH value (± SD)	Refractive index (nD ± SD)
Cleansing gel A	5.34 ± 0.02	1.3522 ± 0.0002
Cleansing gel B	5.20 ± 0.01	1.3515 ± 0.0001
Serum A	5.15 ± 0.02	1.3442 ± 0.0001
Serum B	5.20 ± 0.01	1.3423 ± 0.0001
Face cream A	5.13 ± 0.01	1.3579 ± 0.0003
Face cream B	5.19 ± 0.02	1.3421 ± 0.0001

#### 2.3.2. Particle size distribution (PSD) analysis by laser diffraction (LD).

**Fig 1:**
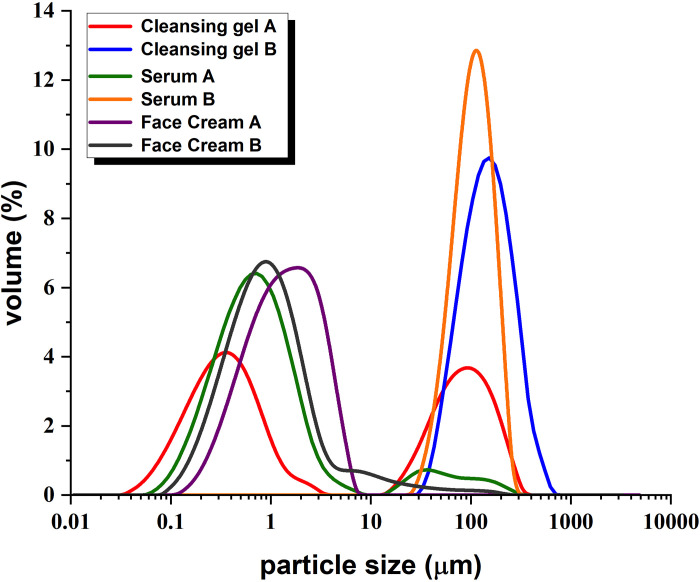
Particle size distribution of each formulation obtained containing apple pomace extract.

**Fig 2:**
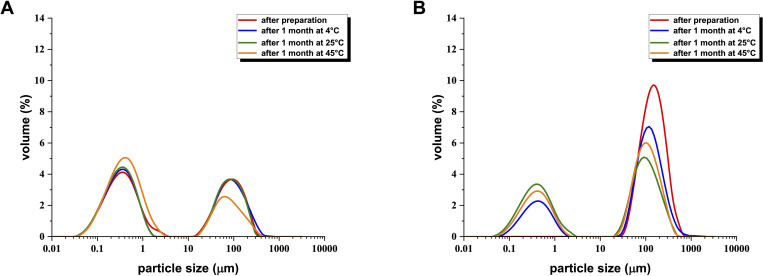
Particle size distribution of Cleansing gels A and B with apple pomace extract.

**Fig 3:**
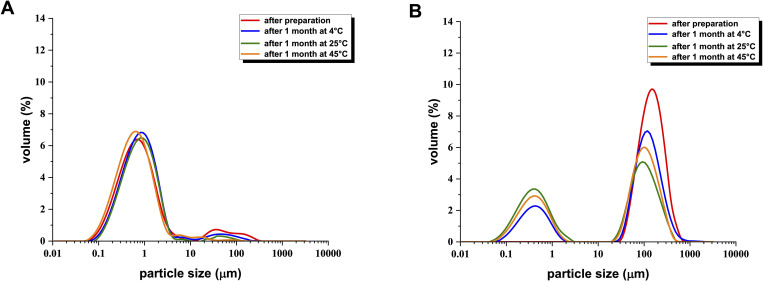
Particle size distribution of Serums A and B with apple pomace extract.

**Fig 4:**
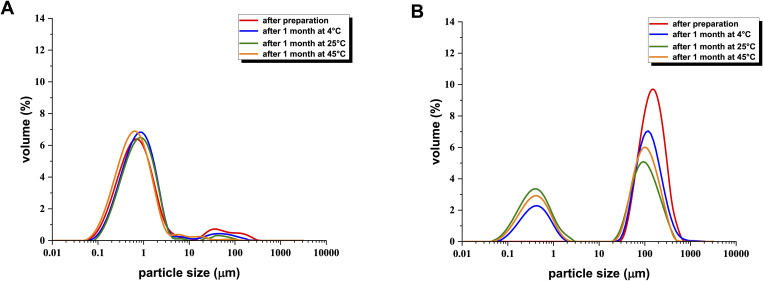
Particle size distribution of Face creams A and B with apple pomace extract.

The particle size distribution (PSD) of formulations containing apple pomace extract was assessed using a Mastersizer 3000 (Malvern Instruments Ltd., Malvern, UK). Prior to the measurements, the refractive index of the formulations was determined using a refractometer (InsMark Instrument Technology Co. Ltd., Shanghai, China) (Table 4.). Samples containing apple pomace were dispersed in distilled water at room temperature and introduced into the optical unit. The measurements were conducted five times and are presented in Table 5 and in Fig 1 (all formulations), Fig 2 (cleansing gels), Fig 3 (serums) and Fig 4 (face creams) [37,38].

**Table 5:** Particle size distribution of all formulations**.

Cleansing Gel A	G_A_ first day (mean ± SD)		G_A_ after one month 4°C (mean ± SD)		G_A_ after one month 25°C (mean ± SD)		G_A_ after one month 45°C (mean ± SD)	
ultrasound	no	yes	no	yes	no	yes	no	yes
D [4.3]	43.76 ± 1.15 μm	40.16 ± 1.16* μm	48.30 ± 1.36 μm	43.50 ± 0.94* μm	41.32 ± 3.72 μm	34.50 ± 1.53* μm	28.20 ± 2.39 μm	24.70 ± 1.66* μm
*p (no/yes)*	*p = 0.0012*		*p = 0.0002*		*p = 0.0053*		*p = 0.0275*	
	p < 0.0001, F = 89.45							
d(10)	0.15 ± 0.00 μm	0.15 ± 0.00 μm	0.15 ± 0.00 μm	0.15 ± 0.00 μm	0.15 ± 0.00 μm	0.14 ± 0.00 μm	0.15 ± 0.00 μm	0.15 ± 0.00 μm
d(50)	0.86 ± 0.01 μm	0.82 ± 0.02 μm	0.76 ± 0.04 μm	0.68 ± 0.01 μm	0.71 ± 0.08 μm	0.60 ± 0.02 μm	0.60 ± 0.02 μm	0.57 ± 0.01 μm
d(90)	140.00 ± 2.45 μm	135.80 ± 2.45 μm	150.00 ± 2.70 μm	140.80 ± 2.17 μm	133.00 ± 8.88 μm	120.00 ± 3.36 μm	101.00 ± 7.20 μm	89.80 ± 5.33 μm
**Cleansing Gel B**	**G**_**B**_ **first day (mean ± SD)**		**G**_**B**_ **after one month 4°C (mean ± SD)**		**G**_**B**_ **after one month 25°C (mean ± SD)**		**G**_**B**_ **after one month 45°C (mean ± SD)**	
D [4.3]	168.00 ± 32.06 μm	88.70 ± 1.05* μm	111.12 ± 24.54 μm	83.40 ± 2.31* μm	77.30 ± 8.15 μm	66.96 ± 2.13* μm	79.88 ± 12.85 μm	64.76 ± 0.79* μm
p (no/yes)	*p = 0.0006*		*p = 0.0361*		*p = 0.0253*		*p = 0.0304*	
p < 0.0001, F (7,32) = 24.27								
d(10)	66.96 ± 5.77 μm	0.35 ± 0.02 μm	0.32 ± 0.05 μm	0.26 ± 0.01 μm	0.23 ± 0.03 μm	0.21 ± 0.00 μm	0.25 ± 0.03 μm	0.22 ± 0.00 μm
d(50)	148.00 ± 22.80 μm	84.38 ± 0.65 μm	89.82 ± 16.78 μm	65.88 ± 1.58 μm	57.90 ± 10.34 μm	44.64 ± 0.98 μm	63.70 ± 14.89 μm	46.36 ± 0.57 μm
d(90)	301.60 ± 73.01 μm	184.60 ± 2.61 μm	246.00 ± 38.78 μm	202.20 ± 5.07 μm	197.80 ± 12.09 μm	180.00 ± 4.42 μm	197.60 ± 15.79 μm	174.00 ± 2.55 μm
**Serum A**	**S**_**A**_ **first day (mean ± SD)**		**S**_**A**_ **after one month 4°C (mean ± SD)**		**S**_**A**_ **after one month 25°C (mean ± SD)**		**S**_**A**_ **after one month 45°C (mean ± SD)**	
D [4.3]	7.70 ± 0.56 μm	5.37 ± 0.27* μm	4.16 ± 0.41 μm	2.33 ± 0.92* μm	4.53 ± 0.52 μm	1.60 ± 0.21* μm	3.80 ± 1.22 μm	1.48 ± 0.75* μm
p (no/yes)	*p < 0.0001*		*p = 0.0036*		*p < 0.0001*		*p = 0.0068*	
p < 0.0001, F (7,32) = 46.55								
d(10)	0.22 ± 0.01 μm	0.19 ± 0.00 μm	0.26 ± 0.02 μm	0.21 ± 0.01 μm	0.26 ± 0.03 μm	0.19 ± 0.00 μm	0.25 ± 0.03 μm	0.20 ± 0.01 μm
d(50)	0.72 ± 0.02 μm	0.62 ± 0.01 μm	0.80 ± 0.05 μm	0.68 ± 0.03 μm	0.81 ± 0.07 μm	0.59 ± 0.00 μm	0.79 ± 0.07 μm	0.61 ± 0.03 μm
d(90)	4.23 ± 0.36 μm	2.43 ± 0.05 μm	2.51 ± 0.07 μm	2.19 ± 0.13 μm	2.75 ± 0.11 μm	1.82 ± 0.05 μm	2.38 ± 0.09 μm	1.85 ± 0.14 μm
**Serum B**	**S**_**B**_ **first day (mean ± SD)**		**S**_**B**_ **after one month 4°C (mean ± SD)**		**S**_**B**_ **after one month 25°C (mean ± SD)**		**S**_**B**_ **after one month 45°C (mean ± SD)**	
D [4.3]	112.40 ± 1.52 μm	99.46 ± 12.18* μm	94.78 ± 5.21 μm	86.18 ± 1.55* μm	83.70 ± 2.31 μm	76.96 ± 1.40* μm	81.94 ± 1.68 μm	73.80 ± 2.80* μm
p (no/yes)	*p = 0.0462*		*p = 0.0076*		*p = 0.0005*		*p = 0.0005*	
p < 0.0001, F (7,32) = 33.20								
d(10)	55.90 ± 0.26 μm	54.27 ± 0.21 μm	0.76 ± 0.01 μm	0.79 ± 0.02 μm	0.71 ± 0.01 μm	0.66 ± 0.01 μm	0.59 ± 0.01 μm	0.54 ± 0.02 μm
d(50)	105.80 ± 1.10 μm	95.70 ± 9.12 μm	85.70 ± 4.45 μm	76.64 ± 1.92 μm	75.16 ± 2.21 μm	67.40 ± 2.18 μm	68.42 ± 2.94 μm	57.30 ± 3.60 μm
d(90)	180.40 ± 2.70 μm	171.00 ± 3.61 μm	203.80 ± 9.36 μm	190.80 ± 2.39 μm	188.20 ± 4.44 μm	176.00 ± 1.22 μm	194.40 ± 5.22 μm	184.00 ± 5.39 μm
**Cream A**	**C**_**A**_ **first day (mean ± SD)**		**C**_**A**_ **after one month 4°C (mean ± SD)**		**C**_**A**_ **after one month 25°C (mean ± SD)**		**C**_**A**_ **after one month 45°C (mean ± SD)**	
D [4.3]	1.16 ± 0.01 μm	1.18 ± 0.00* μm	1.96 ± 0.01 μm	1.96 ± 0.01 μm	1.89 ± 0.01 μm	1.76 ± 0.08* μm	1.78 ± 0.03 μm	1.71 ± 0.04* μm
*p (no/yes)*	*p = 0.0021*			*p = 1.0000*	*p = 0.0069*		*P = 0.0140*	
p < 0.0001, F (7,32) = 453.7								
d(10)	0.28 ± 0.00 μm	0.29 ± 0.00 μm	0.43 ± 0.01 μm	0.43 ± 0.00 μm	0.41 ± 0.00 μm	0.39 ± 0.02 μm	0.48 ± 0.03 μm	0.48 ± 0.01 μm
d(50)	0.91 ± 0.01 μm	0.94 ± 0.00 μm	1.40 ± 0.01 μm	1.40 ± 0.01 μm	1.36 ± 0.00 μm	1.33 ± 0.03 μm	1.38 ± 0.05 μm	1.38 ± 0.02 μm
d(90)	2.44 ± 0.01 μm	2.46 ± 0.00 μm	3.62 ± 0.01 μm	3.62 ± 0.01 μm	3.50 ± 0.01 μm	3.37 ± 0.08 μm	3.32 ± 0.02 μm	3.21 ± 0.06 μm
**Cream B**	**C**_**B**_ **first day (mean ± SD)**		**C**_**B**_ **after one month 4°C (mean ± SD)**		**C**_**B**_ **after one month 25°C (mean ± SD)**		**C**_**B**_ **after one month 45°C (mean ± SD)**	
D [4.3]	3.20 ± 0.24 μm	1.47 ± 0.07* μm	4.35 ± 0.79 μm	2.04 ± 0.33* μm	3.54 ± 0.35 μm	1.97 ± 0.39* μm	1.51 ± 0.01 μm	1.42 ± 0.04* μm
*p (no/yes)*	*p < 0.0001*		*p = 0.0003*		*p = 0.0002*		*p = 0.0012*	
p < 0.0001, F (7,32) = 46.19								
d(10)	0.28 ± 0.00 μm	0.28 ± 0.00 μm	0.28 ± 0.01 μm	0.29 ± 0.00 μm	0.29 ± 0.02 μm	0.29 ± 0.00 μm	0.38 ± 0.00 μm	0.36 ± 0.01 μm
d(50)	0.89 ± 0.00 μm	0.83 ± 0.01 μm	0.87 ± 0.05 μm	0.88 ± 0.00 μm	0.89 ± 0.00 μm	0.88 ± 0.01 μm	1.07 ± 0.00 μm	1.02 ± 0.02 μm
d(90)	3.45 ± 0.06 μm	2.44 ± 0.07 μm	4.16 ± 0.52 μm	2.82 ± 0.23 μm	3.21 ± 0.09 μm	2.65 ± 0.17 μm	2.72 ± 0.00 μm	2.59 ± 0.06 μm

* Statistically significant differences for p < 0.05 vs no ultrasound variant.

**d(0.1) (μm) – 10% of the particles have smaller size than this value; d(0.5) (μm) – median; d(0.9) (μm) – 90% of the particles have smaller size than 0.9 μm, D [3,4] mean diameter based on volume-weighted.

#### 2.3.3. Stability test by multiple light scattering.

The stability measurements of formulations containing apple pomace extract were conducted directly after the preparation of each cosmetic product and at different times for 30 days by MultiScan MS 20 (DataPhysics Instruments GmbH, Germany). Multiple light scattering method was used to measure the stability of the formulations with apple pomace extract at 4°C, 25°C, and 45°C within one month.

Prior to analysis, the samples were gently mixed to ensure homogeneity while avoiding air incorporation. Approximately 10 mL of each formulation was transferred using a syringe into cylindrical glass cells with a total volume of 20 mL. Care was taken to avoid the formation of air bubbles during sample preparation. The external surfaces of the cells were cleaned before insertion into the measurement chamber.

**Fig 5:**
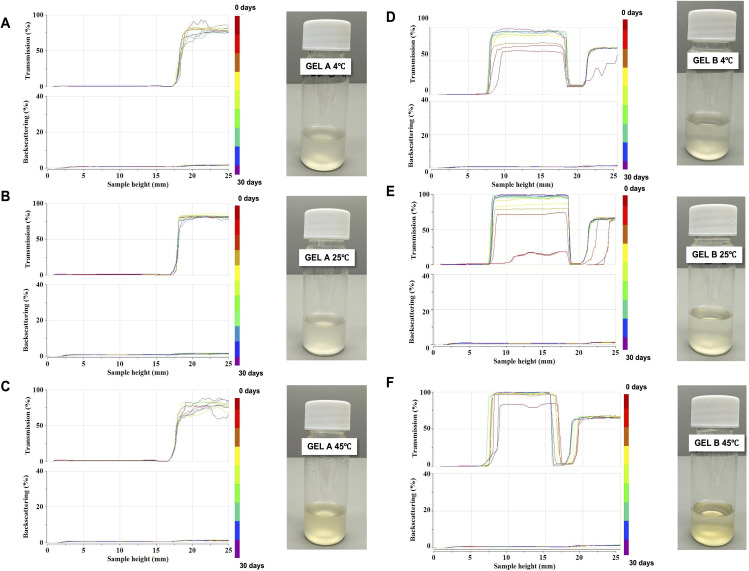
Transmission and Backscattering profiles of Cleansing gels A (A-C) and B (D-F) stored at different temperature conditions.

**Fig 6:**
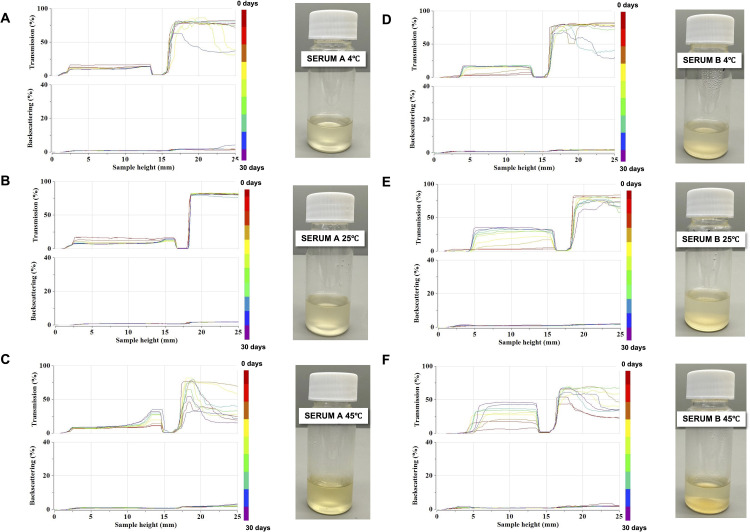
Transmission and Backscattering profiles of Serums A (A-C) and B (E-F) stored at different temperature conditions.

**Fig 7:**
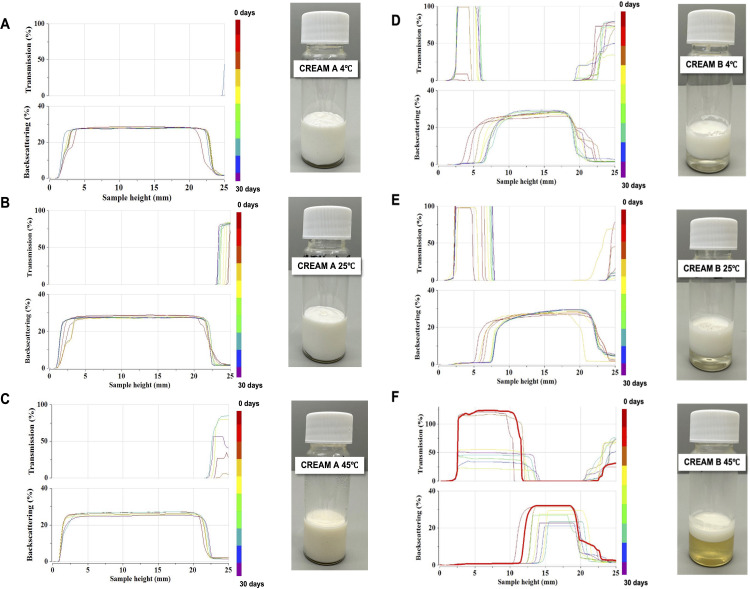
Transmission and Backscattering profiles of Face cream A (A-C) and B (D-F) stored at different temperature conditions.

The results were presented as plots of the intensity of backscattered and transmitted light versus the sample height (Figs 5–7). Based on the obtained data formulation stability changes such as changes in particle size (e.g.,: coalescence) or particle migration (e.g.,: creaming) can be observed and identified. Due to the opacity of all samples, their stability was evaluated by analysing variations in the backscattering (BS) profiles at temperatures corresponding to storage conditions (4°C, 25°C, and 45°C). Reference BS profiles were collected immediately after the preparation of the formulations [39,40].

#### 2.3.4. Microscopic evaluation of the formulations.

Microscopic evaluation was carried out immediately after preparing the formulations and after 30 days of storage under various temperature conditions (4°C, 25°C, and 45°C) by the Digital Light Microscope Keyence equipped with a VH-Z100R variable focus lens.

A small amount of the formulation (approximately 10–20 mg) was placed on a glass microscope slide and gently spread to obtain a thin, uniform layer. The sample was covered with a coverslip and immediately analysed. The microscopic image is intended to illustrate the stability changes occurring in the samples (Fig 9).

#### 2.3.5. Statistical analysis.

Statistical analyses for the results of particle size distribution and stability indices were carried out using the MANOVA Friedman multivariate analysis of variance test for paired data. The assumptions of normality and homogeneity of variances were verified prior to MANOVA. The data met the criteria for the applied tests. Statistical significance was tested using Dunn’s post-hoc test. The data are shown as the mean values ± SD. All the relevant test probability values (p < 0.05) are included in Table 5.

## 3. Results and discussion

### 3.1. Characterization of cosmetic formulations

In these studies, two different types of cleansing gel, serum, and face cream enriched with apple pomace extract were used. According to information provided by the producer the recommended concentration of the ingredient in formulations is 1–3%. Accordingly, the prepared formulations contain apple pomace extract within this range. Cleansing gels have the lowest concentration among the three types of cosmetics, containing 1% w/w apple pomace extract, as they have the shortest contact time with the skin and are rinse-off formulations. The next highest concentration, 2% w/w, is used in face creams, which are leave-on products, while the highest concentration, 3% w/w, is applied in serums. As per their definition, serums are characterized by the highest concentration of active ingredients among all skincare steps in this set. Moreover, the ingredient is standardized for its polyphenol content (minimum 300 ppm), ensuring consistent quality and efficacy across the formulations. The pH and the refractive index values of the formulations were analysed, and the results are presented in Table 4. The pH values were adjusted to a range of 5.0–5.5 to ensure formulations suitable for facial skin, while also optimizing the efficacy of preservatives (Euxyl K712, Euxyl PE9010, and Euxyl K903) in accordance with the manufacturer’s specifications. According to standards, the pH value of topical formulations should be between 4 and 6 [41]. The measured pH values of the formulations in this study complied with these standard requirements. The pH values of all the cosmetic formulations were within the range of 5.15–5.20, aligning with the natural pH of the skin and ensuring compatibility and safety for topical application. This stability in pH indicates that the formulations are unlikely to cause irritation or disrupt the skin’s acid mantle.

The refractive indices of formulations were determined to be used in particle size distribution (PSD) measurements. The refractive index value for cosmetic hydrogels depends on their chemical composition and application, typically ranging between 1.33 (the refractive index of water, the main component of many hydrogels) and 1.50 (characteristic of higher optical density ingredients like gelling agents, humectants such as glycerine, or active substances) [42]. The refractive index value of face cream A was characteristic of an oil-in-water emulsion [43].

Fig 1 presents the particle size distribution curves of each formulation directly after their preparation. The general characteristic of the droplet size distribution measurements was also gathered in Table 5. Depending on the type of cosmetic preparation the results were different. The largest particle size in the range from 50 μm to 1000 μm was observed for Cleansing gel B while the smallest particles were detected for Cleansing gel A which had bimodal particle size distribution with two different and controlled droplet sizes. A monomodal particle size distribution (PSD) was generated for most formulations obtained (Cleansing gel B, Serum A, Serum B, and Face cream A). As it is generally known various parameters influence the particle size of emulsions. An important role plays a surfactant type and its concentration [44]. By adding the appropriate emulsifier, the particle size distribution in the emulsion can be controlled. This phenomenon was observed in the case of Face cream A and Face cream B. It was detected that the presence of Olivem 1000 (Cetearyl Olivate, Sorbitan Olivate) in Face cream A had an impact on the reduction of particle size of this formulation compared to Face cream B which was prepared without adding this emulsifier [45]. The tighter distribution curve and more uniform particle sizes were observed for Face cream A compared to Face cream B which is desirable in the formulation development.

### 3.2. Stability studies of formulations containing apple pomace extract by laser diffraction

The laser diffraction method was also used to monitor the stability of formulations. The variations in particle size distribution within the cosmetic preparation over storage time is the initial indicator of its aging [46]. Investigating the droplet size is a crucial step in optimizing the formulations’ stability, safety, and performance. Droplet size analysis results are usually shown on a histogram. Figs 3–5 present the particle size distribution of gels, serums, and emulsions containing apple pomace extract. Additionally, in Table 5 the d(0.1), d(0.5), d(0.9) and volume mean diameter D[4,3] were gathered. The high D[4,3] values indicate the presence of destabilization processes in the formulation [47] It was observed that Cleansing gel A was more stable than Cleansing gel B. There were no significant differences in the particle sizes of gel A directly after preparation and after one-month of storage at various temperature conditions. In each case the distribution curves were bimodal. For gel B the particle size distribution after preparation was monomodal and after time storage at 4°C, 25°C, and 45°C the distribution curves changed to bimodal. Moreover, much higher D[4,3] values were observed for Cleansing gel B on the preparation day and after 30 days of storage at different temperature conditions compared to the results obtained for Cleansing gel A. The volume mean diameter of untreated Cleansing gel B directly after preparation was 168.0 μm and decreased to 88.7 μm after ultrasound treatment. While for Cleansing gel A the D[4,3] remained practically the same following ultrasound treatment. It means that Guar Hydroxypropyltrimonium Chloride (N-Hance 3196) applied in gel A was a much better gelling agent providing higher stability compared to Cyamopsis Gum Tetragonoloba (Guar gum) used in gel B. Guar gum being a natural polymer may undergo a controlled degradation while subjected to sonication. Furthermore, it was reported that the application of ultrasound on the mixtures of carbohydrates can result in depolymerisation caused by the mechanical and chemical processes linked with cavitation [48,49]. Cavitational thermolysis can generate hydroxyl radicals and hydrogen atoms that can form hydrogen peroxide or even hydroperoxyl radical when oxygen is not present [50]. These short-lived reactive molecules can then interact with carbohydrates. This phenomenon could have occurred in the case of gel B that was subjected to ultrasound treatment.

Similar observations were detected for serums. When guar gum was applied as a gelling agent the serum was less stable after one month of storage at different temperature conditions compared to Serum A. No meaningful changes in particle size distribution after time were detected in Serum A, which contained xanthan gum as a stabilizer. This highlights that guar gum has inferior properties as a gelling agent compared to xanthan gum, as it loses viscosity under extreme pH conditions and at elevated temperatures [51]. The average particle size D[4.3] of Serum B stored 30 days at room temperature decreased from 81.9 μm to 2.24 μm after ultrasound treatment. It proves that high intensity sonification can affect both the structural and functional properties of polysaccharides [52]. Tiwari et al. reported significantly higher changes in rheological properties of guar gum dispersions subjected to sonication than that of xanthan gum [53].

On the other hand, the addition of natural emulsifier such as Olivem 1000 in Face cream A had an impact on its better stability at different storage conditions compared to Face cream B which did not contain this ingredient. Olivem 1000 acts as a multifunctional emulsifier and stabilizer, enhancing the structural integrity of emulsions, preventing phase separation, and improving overall product stability. In Face cream A, it played a key role in maintaining consistency and homogeneity under different storage conditions. For stable emulsions histogram shows a single peak that represents one population of droplets [54]. The small droplets with uniform size in a narrow range (0.1–10 μm) were observed for Face cream A that was stored for 30 days at different temperature. Furthermore, D [3,4] mean diameter based on volume-weighted remained unchanged after ultrasound treatment in each tested condition. In contrast, in Face cream B two populations of droplets one in range from 0.1–10 μm and the other one in range from 10 to 150 μm were detected. Additionally, the value of D [4.3] decreased from 3.20 μm to 1.47 μm after ultrasound treatment that indicates the instability of this system.

The statistical analyses were performed to indicate the influence of ultrasound treatment on particle size distribution of each formulation. All results, except for Cream A after one month in 4°C, demonstrated statistically significant differences for p < 0.05 vs no ultrasound variants.

### 3.3. Stability studies of formulations containing apple pomace extract by multiple light scattering

The advanced optical method, such as multiple light scattering was applied to determine the stability of the formulations obtained. This technique allows the identification of processes in colloidal systems such as flocculation, creaming, sedimentation, and phase separation [55]. In MLS technique the entire sample height is scanned using near-infrared light to detect and record even minor changes within the emulsion. The main advantage of this method is the possibility to analyse the sample without dilution [46], which occurred during the assessment of particle size distribution. The results were presented as intensity of transmission (T) and backscattering (BS) versus sample height in Figs 5–7. The curve at 0 days (start of the analysis) is red and gradually changes to purple at day 30 (end of the experiment). The sample is considered as stable where no changes in BS and T occurred. In Fig 5 A, B,C it was observed that for Cleansing gel A regardless of the temperature conditions both BS and T profiles remained constant over one month of storage.

The light beam could not pass through the cell containing Cleansing gel A resulting in a low transmission intensity and the formation of a well-dispersed suspension. According to Goscianska and Olejnik [56] the lower the transmission levels, the greater the stability of dispersion. These results indicated the high stability of Cleansing gel A in three different temperature ranges. In contrast for Cleansing gel B the variations in transmission profiles were observed at 4, 25 and 45 °C (Fig 5 D,E,F). The transmission intensity in each case increased within the 30 days of analysis. It means that Cleansing gel B was sensitive to temperature conditions and was not stable.

In the case of both serums the transmission curves were analysed because the backscattering intensity was low (Fig 6). At 4°C and 25°C the intensity of transmission profiles of Serum A (Fig 6 A,B) changed only slightly. Whereas at 45°C the intensity increased slightly towards the top of the vial over time. In turn, significantly higher variations in transmission intensity were noted in the case of Serum B. The temperature conditions had an impact on the stability of this sample. The transmission increased with the change rate of 0.49%/d at 4°C, 0.83%/d at 25°C and 1.04%/d at 45°C. The most significant differences were detected at elevated temperature. The transmission intensity increased from 8% to 45% when the sample was stored at 45°C.

The results of stability studies of face creams were presented in Fig 7. In the case of Face cream A, no signal was detected in the transmission profile because the sample was opaque, and no phase separation was observed under any of the tested temperature conditions. After 30 days of storage, no significant changes in backscattering intensity were observed. In contrast, full phase separation was identified for Face cream B at all temperature conditions. At the lower part of the vial the sample was transparent, so the transmission intensity was 100%. The greatest changes in stability were observed at 45 °C. With time, the transmission band shifted to higher sample heights and a decrease in backscattering intensity was observed Additionally, the stability index was computed to enhance visualization of sample changes and to perform the overall analysis of tested cosmetic formulations.

**Fig 8:**
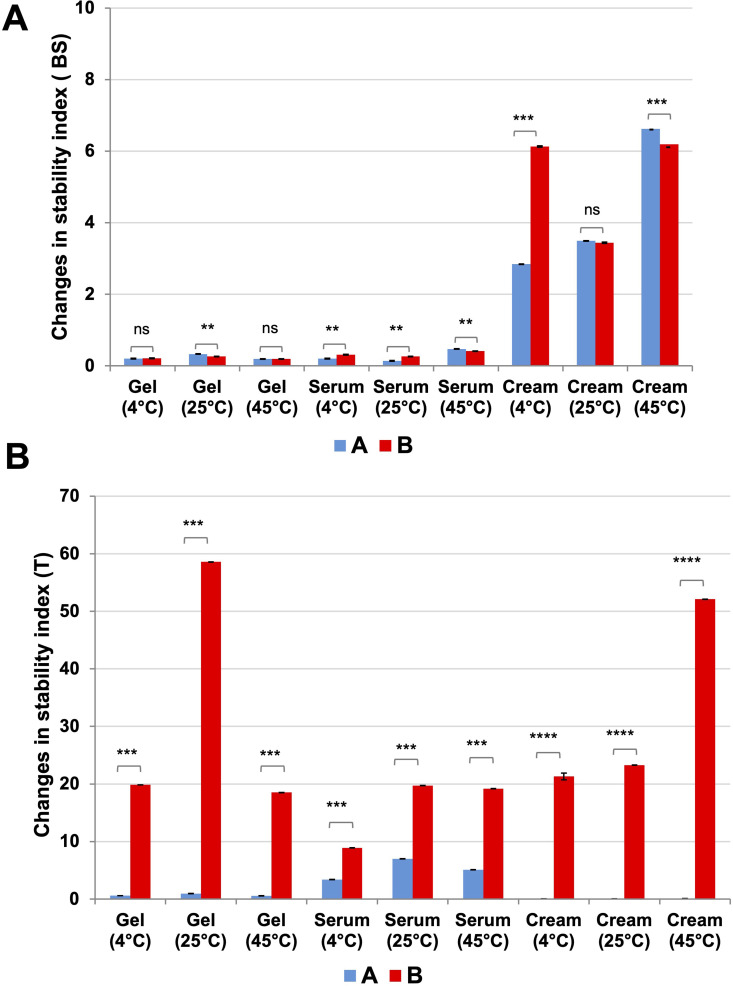
Stability changes after one month of storage (Statistical significance was set at p < 0.05. Significance levels are indicated as follows: ns (not significant), * p < 0.05, ** p < 0.01, *** p < 0.001, **** p < 0.0001).

This index sums all the variations occurring in transmission and backscattering and enables to compare the stability of topical preparations. It should be highlighted that the sample is considered as unstable if the variation of backscattering exceeds 10% [57]. Consequently, the smaller the changes in the sample, the greater its stability. According to Lemarchand et al. [58] analysing the BS signal is only possible when the T signal is absent. If not, the partial reflection of the light passing through the sample by the measurement cell walls would disrupt the BS signal. Therefore, the changes in stability index were presented separately for backscattering and transmission (Fig 8). It can be clearly seen that in each type of formulation samples marked as A were much more stable. In the case of gel A the changes in stability index for both transmission and backscattering at three different temperature conditions were very low after one month. Whereas the results of changes in transmission of gel B were high in each environment. Much higher T-SI value was detected for Serum B compared to Serum A. In the case of Face cream A only variation in BS-SI was considered because no phase separation was detected. In turn, in Face cream B high T-SI was observed that proved its low stability. For each pair of formulations A and B, we calculated p-values to confirm whether statistically significant differences exist between samples. Detailed numerical data of p-values have also been added to the supplementary materials (Table S4 in S1 File).

It should be highlighted that multiple light scattering analysis demonstrated that no significant variations in Backscattering and Transmission profiles of Cream A, Cleansing Gel A, or Serum A occurred during the long-term storage period, indicating that all formulations maintained their physical integrity and homogeneity over six months. The results of these analyses are presented in the Supporting Information (Fig. S5 and S6 in S1 File).

Furthermore, the consideration must be given to the fact that the presence of apple pomace (AP) extract, containing polyphenols, organic acids, and natural pigments, may potentially influence light scattering and absorption during MLS and LD analyses. Therefore, to evaluate the impact of natural plant-derived ingredients on formulation performance and measurement reliability, control systems without extract were developed alongside the cosmetics samples (see Supporting Information Table S1-S3 in S1 File). Advanced stability techniques (MLS and optical microscopy) demonstrated that the reference formulations without extract (Gel A, Serum A, Cream A) remained structurally stable under storage conditions, whereas the intentionally destabilized variants (Gel B and Cream B) exhibited predictable instability phenomena, such as phase separation (see Supporting Information Fig. S1-S4 in S1 File). Upon incorporation of the apple pomace extract, the stable matrices (Gel A, Serum A, Cream A) preserved their structural integrity, whereas less robust systems (Gel B, Serum B, Cream B) displayed accelerated instability under thermal stress. Importantly, Serum A and Serum B without AP extract were transparent and colorless throughout the study period. Although the Serums presented in this study contained the highest extract loading (3% w/w), resulting in the most pronounced shift to a yellowish hue due to polyphenols and associated chromophores, this coloration is inherent to the apple pomace extract, as stated by the supplier, and therefore does not indicate instability. The pigmentation introduced a measurable but minor optical absorption effect, along with a likely slight increase in refractive index; however, these shifts did not generate artificial scattering artifacts. In Serum A, where no physical destabilization was observed, MLS/LD profiles remained consistent despite the color change, confirming that the extract did not compromise the accuracy of particle size distribution measurements. In contrast, for Serum B and Cream B, in which MLS suggested structural evolution, optical microscopy corroborated the presence of droplet growth and particle redistribution, demonstrating genuine physical instability rather than optical interference.

These findings indicate that although the apple pomace extract (particularly at the highest concentration used) may slightly modify optical properties of the formulations, the combined analytical approach reliably differentiated between matrix-related optical effects and genuine structural changes. Additionally, visual inspection of stable formulations (A variants) showed no precipitation or turbidity increase during storage, suggesting that the apple pomace extract remained well-dispersed and did not form separate light-scattering phases. Therefore, while the natural components of extracts could theoretically influence optical signals, our multi-technique and cross-validated evaluation confirmed that such matrix effects were negligible and did not compromise the measurement accuracy. Thus, natural ingredients such as apple pomace extract (at concentrations used in presented formulations) did not impair the validity of MLS and LD-based stability assessments, and these advanced scattering techniques remain suitable for analyzing cosmetics containing plant-derived products.

### 3.4. Microscopic evaluation of the formulations

**Fig 9:**
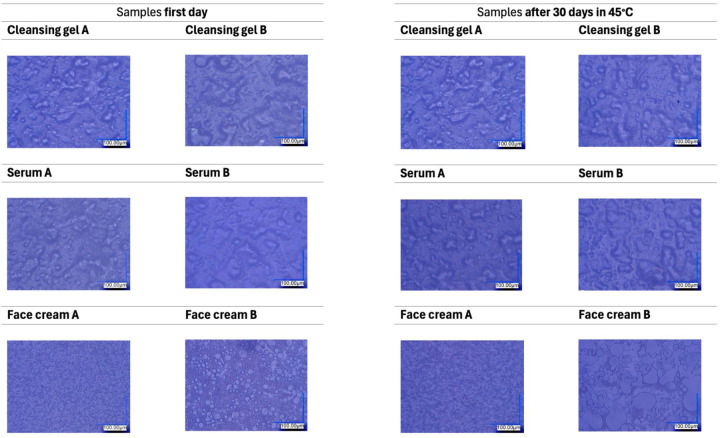
Microscopic images of cleansing gels, serums and face cream stored one month at 45°C.

Microscopic images illustrate the six samples (Cleansing gel A, Cleansing gel B, Serum A, Serum B, Face cream A, Face cream B) directly after preparation and after one month of storage at 45°C. In the case of cleansing gels and serums the irregularly shaped droplets were observed regardless of the temperature conditions (Fig 9). It was difficult to detect the changes in visual stability after time using optical microscopy for hydrogels. While it was observed that the droplets of Face cream A were homogenously distributed and were relatively uniform in size. The morphology of the droplets in Face cream A did not change after 30 days of storage at three different temperature conditions. While in the microscope image of Face cream B small and large droplets were detected at the same time which suggests that the separation process might occur through coalescence of Ostwald ripening [36]. The optical microscope images of Face creams A and B presented in Fig 9 are consistent with measurements performed using multiple light scattering method.

### 3.5. Scalability considerations

Although our experiments were performed at laboratory scale, industrial manufacture may introduce variations in mixing and homogenization dynamics, thermal gradients, and raw-material consistency. Accordingly, scale-up will prioritize geometric similarity, power-per-volume (or tip-speed) matching, controlled heating and cooling profiles, and specification-based standardization of the apple-pomace extract, supported by in-line monitoring of key parameters such as particle size distribution, viscosity, and pH. These measures aim to ensure consistent microstructure and uniform distribution of functional components and preservatives throughout bulk production and high-throughput filling operations.

## 4. Conclusions

This study demonstrated the successful development and stability assessment of cosmetic formulations incorporating extract from apple waste, including cleansing gels, serums, and face creams, prepared in stable and unstable variants. By leveraging advanced stability testing techniques —multiple light scattering, laser diffraction and optical microscopy—the research highlighted the potential of these methods to detect even subtle changes in formulation stability early in the development process. These findings emphasize their utility for optimizing cosmetic formulations, ensuring product safety, efficacy, and market readiness.

The use of apple pomace extract, an upcycled by-product rich in bioactive compounds, highlights its potential to support sustainable and eco-friendly practices in the cosmetic industry. This study further demonstrated how even minor alterations, such as changing or removing a single formulation component, can have a significant impact on product stability, providing valuable insights into the specific roles of individual ingredients. A detailed analysis revealed substantial variations in stability depending on the type of agents used, with clear differences observed across the cleansing gels, serums, and face creams.

- Cleansing Gel: The use of N-Hance 3196 (Guar Hydroxypropyltrimonium Chloride) in Cleansing Gel A resulted in higher stability compared to Cleansing Gel B, which utilized Guar gum (Cyamopsis Gum Tetragonoloba). Cleansing Gel A maintained a proper particle size distribution over time and across different storage temperatures, whereas Cleansing Gel B showed a shift in particle size distribution, indicating reduced stability.
- Serum: The inclusion of xanthan gum in Serum A significantly improved its stability, as evidenced by the lack of meaningful changes in particle size distribution during storage. Conversely, Serum B, which utilized Guar gum (Cyamopsis Gum Tetragonoloba) as a gelling agent exhibited reduced stability under the same conditions.
- Face cream: The incorporation of Olivem 1000 (Cetearyl Olivate, Sorbitan Olivate) in Face cream A contributed to a more uniform particle size distribution and tighter stability profiles compared to Face cream B, which lacked this emulsifier. This difference underscores the critical role of emulsifiers in enhancing stability, particularly for formulations stored at elevated temperatures.

The analysis of cosmetics enriched with apple pomace proved that changing only one component in the sample can influence the stability of formulations. Based on the results obtained by multiple light scattering it was clearly seen that in the case of creams the addition of Olivem 1000 (that serves as an oil-in-water emulsifier) significantly improved the stability of the formulation. Phase separation occurred in the absence of Olivem 1000 in the case of Face cream B. The multiple light scattering and laser diffraction methods were both effective for assessing the stability of emulsions enriched with apple pomace extract. However, it should be highlighted that the sample in MLS is analysed in its natural and undiluted state, therefore this method is more reliable. In contrast, LD involves diluting and mixing the sample, which can alter its properties. Optical microscopy is also an effective method for observing changes in the tested emulsions, as it clearly reveals this phenomenon. On the other hand, optical microscopy was not effective in detecting changes in the tested hydrogel samples. Whereas both MLS and LD were appropriate for assessing stability of the designed hydrogel formulations—cleansing gels and serums.

This research also highlighted that advanced stability testing techniques, while predominantly applied in academic research, have significant potential for successful adoption in industrial cosmetic development. The advanced optical methods are able to capture subtle stability variations, making it particularly valuable for both scientific research and industrial applications, offering precise insights into the dynamics of cosmetic formulations. These methods, multiple light scattering, laser diffraction and optical microscopy, offer advantages over traditional approaches by enabling earlier detection of potential instability and providing more detailed insights into formulation behaviour. By adopting these innovative techniques, the cosmetic industry could streamline formulation processes, reduce development timelines, and ultimately enhance product quality. The findings underscore the feasibility of integrating such methods into industrial workflows, bridging the gap between scientific advancements and practical application in manufacturing.

In conclusion, this study highlights the value of integrating sustainable ingredients like apple pomace extract into cosmetics while optimizing stability testing using advanced methods. The findings provide a foundation for further exploration into sustainable practices, cost evaluations, and business strategies for upcycled cosmetic products, ultimately bridging the gap between scientific innovation and practical industrial application.

## Supporting information

S1 File SI1. Preparation of topical formulations without apple pomace extract (Tables S1-S3). SI2. Statistical comparison of changes in stability index. Figure S1. Transmission and Backscattering profiles of Cleansing gels A (A. B) and B (C.D) without apple pomace extract (AP) stored at different temperature conditions. Figure S2. Transmission and Backscattering profiles of Serum A (A. B) and B (C.D) without apple pomace extract (AP) stored at different temperature conditions. Figure S3. Transmission and Backscattering profiles of Cleansing gels A (A. B) and B (C.D) without apple pomace extract (AP) stored at different temperature conditions. Figure S4. Microscopic images of cleansing gels. serums and face cream without apple pomace extract. Figure S5. Transmission and Backscattering profiles of Gel A, Serum A and Cream A stored 6 months. Figure S6. Stability changes after one month and six months of storage.
(ZIP)

S1 Fig Graphical abstract. (TIFF)
