# Supplementary material for: Suitability of stability assessment methods for topical formulations enriched with apple pomace extract
Source: PLoS One. 2026 Jun 24;21(6):e0351678. doi: 10.1371/journal.pone.0351678 (PMC13293396; doi:10.1371/journal.pone.0351678)
Supplement: S1 File — SI2. Statistical comparison of changes in stability index. Figure S1. Transmission and Backscattering profiles of Cleansing gels A (A. B) and B (C.D) without apple pomace extract (AP) stored at different temperature conditions. Figure S2. Transmission and Backscattering profiles of Serum A (A. B) and B (C.D) without apple pomace extract (AP) stored at different temperature conditions. Figure S3. Transmission and Backscattering profiles of Cleansing gels A (A. B) and B (C.D) without apple pomace extract (AP) stored at different temperature conditions. Figure S4. Microscopic images of cleansing gels. serums and face cream without apple pomace extract. Figure S5. Transmission and Backscattering profiles of Gel A, Serum A and Cream A stored 6 months. Figure S6. Stability changes after one month and six months of storage. (ZIP) [file pone.0351678.s001.zip › Fig. S6.docx]

SUPPORTING INFORMATION

**Suitability of stability assessment methods for topical formulations enriched with apple pomace extract**

*Katarzyna Czerniewicz^a.b.*.^ Anna Olejnik^c.d^. Maria Urbańska^b^. Karolina Latanowicz^e^. Justyna Gornowicz Porowska^b^. Krzysztof Kus^a^*

*^a^ Department and Division of Pharmacoeconomics and Social Pharmacy. Poznan University of Medical Sciences. Rokietnicka 3. 60-806 Poznan. Poland. Poland*

*^b^ Department and Division of Practical Cosmetology and Skin Diseases Prophylaxis. Poznan University of Medical Sciences. Rokietnicka 3. 60-806 Poznan. Poland*

*^c^ Faculty of Chemistry. Adam Mickiewicz University in Poznan. Uniwersytetu Poznanskiego 8. 61-614 Poznan Poland*

*^d^ Centre for Advanced Technologies. Adam Mickiewicz University in Poznan. Uniwersytetu Poznanskiego 8. 61-614 Poznan. Poland*

*^e^ Latech Company. Klonowa 2. 62-002 Suchy Las. Poland*

*^*^* Corresponding author:

E-mail: [*kczerniewicz@ump.edu.pl*](mailto:kczerniewicz@ump.edu.pl). +*48792747310* (Katarzyna Czerniewicz)


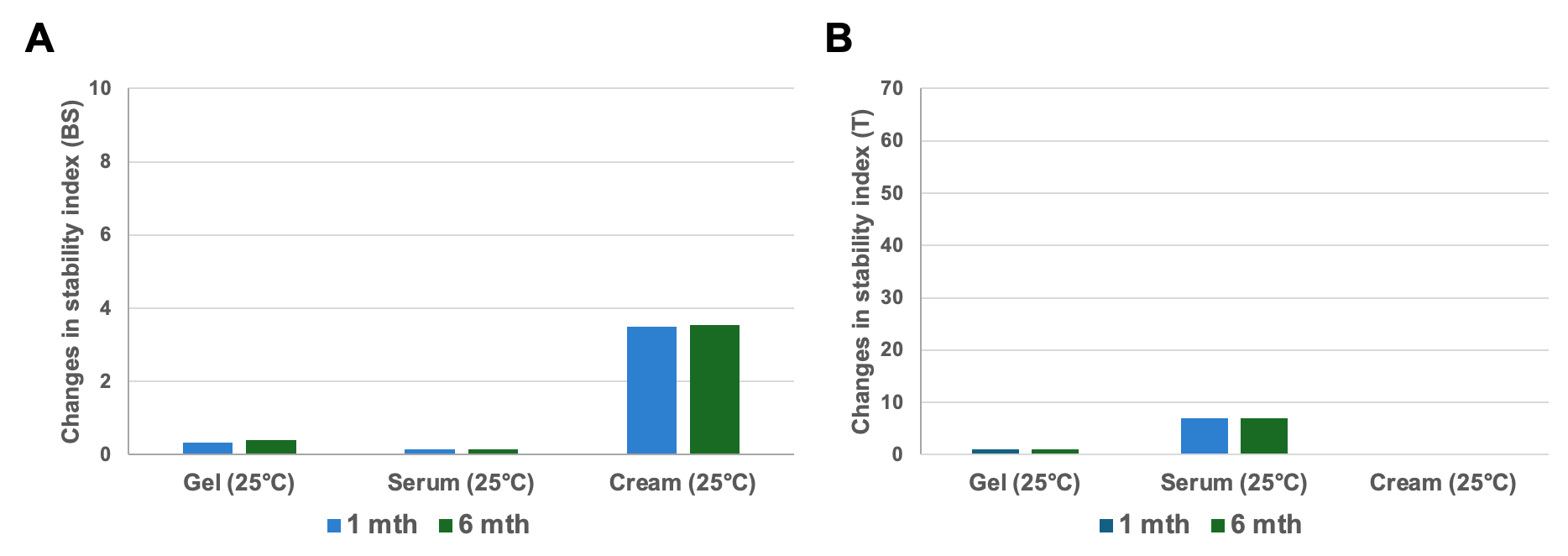


**Figure S6**. Stability changes after one month and six months of storage.
