# Supplementary material for: Suitability of stability assessment methods for topical formulations enriched with apple pomace extract
Source: PLoS One. 2026 Jun 24;21(6):e0351678. doi: 10.1371/journal.pone.0351678 (PMC13293396; doi:10.1371/journal.pone.0351678)
Supplement: S1 File — SI2. Statistical comparison of changes in stability index. Figure S1. Transmission and Backscattering profiles of Cleansing gels A (A. B) and B (C.D) without apple pomace extract (AP) stored at different temperature conditions. Figure S2. Transmission and Backscattering profiles of Serum A (A. B) and B (C.D) without apple pomace extract (AP) stored at different temperature conditions. Figure S3. Transmission and Backscattering profiles of Cleansing gels A (A. B) and B (C.D) without apple pomace extract (AP) stored at different temperature conditions. Figure S4. Microscopic images of cleansing gels. serums and face cream without apple pomace extract. Figure S5. Transmission and Backscattering profiles of Gel A, Serum A and Cream A stored 6 months. Figure S6. Stability changes after one month and six months of storage. (ZIP) [file pone.0351678.s001.zip › SI1.docx]

SUPPORTING INFORMATION

**Suitability of stability assessment methods for topical formulations enriched with apple pomace extract**

*Katarzyna Czerniewicz^a.b.*.^ Anna Olejnik^c.d^. Maria Urbańska^b^. Karolina Latanowicz^e^. Justyna Gornowicz Porowska^b^. Krzysztof Kus^a^*

*^a^ Department and Division of Pharmacoeconomics and Social Pharmacy. Poznan University of Medical Sciences. Rokietnicka 3. 60-806 Poznan. Poland. Poland*

*^b^ Department and Division of Practical Cosmetology and Skin Diseases Prophylaxis. Poznan University of Medical Sciences. Rokietnicka 3. 60-806 Poznan. Poland*

*^c^ Faculty of Chemistry. Adam Mickiewicz University in Poznan. Uniwersytetu Poznanskiego 8. 61-614 Poznan Poland*

*^d^ Centre for Advanced Technologies. Adam Mickiewicz University in Poznan. Uniwersytetu Poznanskiego 8. 61-614 Poznan. Poland*

*^e^ Latech Company. Klonowa 2. 62-002 Suchy Las. Poland*

*^*^* Corresponding author:

E-mail: [*kczerniewicz@ump.edu.pl*](mailto:kczerniewicz@ump.edu.pl). +*48792747310* (Katarzyna Czerniewicz)

**SI. 1. Preparation of topical formulations without apple pomace extract**

**Preparation of topical formulations without apple pomace extract**

Six base formulations—two cleansing gels, two serums and two face creams—were prepared without apple pomace extract in two variants: stable (A) and an intentionally unstable (B). Their qualitative and quantitative compositions are presented in Table S1 (cleansing gels), Table S2 (serums) and Table S3 (face creams).

*Cleansing gel A*

Sodium gluconate (2) was dissolved in distilled water (1), then glycerine (3) and N-Hance 3196 (4) were added. As a next step, the ingredients 6-10 were added. Afterwards, citric acid (11) was introduced until a pH of 5.0-5.5 was reached. The formulation was stirred with an automatic stirrer continuously throughout the whole procedure.

*Cleansing gel B*

Sodium gluconate (2) was solved in distilled water (1), then glycerine (3) and guar gum (5) were added. As a next step, the ingredients , 6-10 were added. Afterwards, citric acid (11) was introduced until a pH between 5.0-5.5 was reached. The formulation was stirred with an automatic stirrer continuously throughout the whole procedure.

*Table S1. Chemical compositions of the base cleansing gels.*

| No | Trade name | INCI | Company | Cleansing Gel A  Quantity  (%, ±0.01) | Cleansing Gel B  Quantity  (%, ±0.01) |
| --- | --- | --- | --- | --- | --- |
| 1 | Water | *Aqua* | – | 80.09 | 81.09 |
| 2 | Sodium gluconate | *Sodium Gluconate* | Brenntag | 0.20 | 0.20 |
| 3 | Glycerine | *Glycerine* | Brenntag | 5.00 | 5.00 |
| 4 | N-Hance 3196 | *Guar Hydroxypropyltrimonium Chloride* | Barentz | 1.30 | - |
| 5 | Guar gum | *Cyamopsis Gum Tetragonoloba* | Barentz | - | 1.30 |
| 6 | Leraamph CAB 35 | *Aqua, Cocamidopropyl Betaine, Sodium Chloride* | Stockmeier | 3.00 | 3.00 |
| 7 | Euxyl K712 | *Sodium Benzoate, Potassium Sorbate, Aqua* | Barentz | 1.50 | 1.50 |
| 8 | Coco glucoside AC818 | *Aqua, Coco Glucoside* | HSH | 7.00 | 7.00 |
| 9 | Apriglu CG-GO 400v2 | *Aqua, Coco-Glucoside, Glyceryl Oleate, Citric Acid* | Stockmeier | 0.50 | 0.50 |
| 10 | Fragrance | *Parfum* | Arcon | 0.20 | 0.20 |
| 11 | Citric acid | *Citric Acid* | Brenntag | 0.21 | 0.21 |

*Serum A*

The ingredients (2-6 and 8-9) were solved in distilled water (1) using an automatic stirrer. Afterwards, citric acid (10) was introduced until a pH between 5.0-5.5 was reached.

*Serum B*

The ingredients (2-5 and 7-9) were added to distilled water (1), then citric acid (10) was introduced until a pH between 5.0-5.5 was reached and stirred until the hydrogel formulation was obtained.

| *Table S2. Chemical compositions of the base serums.*   \| No \| Trade name \| INCI \| Company \| Serum A  Quantity  (% w/w, ±0.01) \| Serum B  Quantity  (% w/w, ±0.01) \| \| --- \| --- \| --- \| --- \| --- \| --- \| \| 1 \| Water \| *Aqua* \| – \| 90.70 \| 93.70 \| \| 2 \| Sodium gluconate \| *Sodium Gluconate* \| Brenntag \| 0.20 \| 0.20 \| \| 3 \| Euxyl K712 \| *Sodium Benzoate, Potassium Sorbate, Aqua* \| Barentz \| 0.50 \| 0.50 \| \| 4 \| Euxyl PE9010 \| *Phenoxyethanol, Ethylhexylglycerin* \| Barentz \| 0.75 \| 0.75 \| \| 5 \| Glycerine \| *Glycerine* \| Brenntag \| 2.00 \| 2.00 \| \| 6 \| Xanthan gum \| *Xanthan gum* \| Brenntag \| 0.70 \| *-* \| \| 7 \| Guar gum \| *Cyamopsis Guma Tetragonoloba* \| Brenntag \| - \| 0.70 \| \| 8 \| Panthenol 75 \| *Panthenol* \| Enzym \| 2.00 \| 2.00 \| \| 9 \| Fragrance \| *Parfum* \| Arcon \| 0.10 \| 0.10 \| \| 10 \| Citric acid \| *Citric acid* \| Brenntag \| 0.05 \| 0.05 \| |
| --- | --- | --- | --- | --- | --- | --- | --- | --- | --- | --- | --- | --- | --- | --- | --- | --- | --- | --- | --- | --- | --- | --- | --- | --- | --- | --- | --- | --- | --- | --- | --- | --- | --- | --- | --- | --- | --- | --- | --- | --- | --- | --- | --- | --- | --- | --- | --- | --- | --- | --- | --- | --- | --- | --- | --- | --- | --- | --- | --- | --- | --- | --- | --- | --- | --- | --- |

*Face cream A*

Distilled water (1) was heated to 70°C and sodium gluconate (2), Euxyl K712 (3), and Euxyl K903 (4) were added. At the same time, the ingredients of the oil phase (5-10) were heated to 70°C. When all the oil phase ingredients were melted, the water phase was added slowly and homogenized. Then the emulsion was cooled below 40°C and ingredients (11, 12) and fragrance (13) were added, and emulsion was homogenized. Afterwards, citric acid (14) was introduced until a pH between 5.0-5.5 was reached and the formulation was homogenized until the proper viscosity was fully developed.

*Face cream B*

Distilled water (1) and the oil phase (6-9) were heated to 70°C separately. To the water phase sodium gluconate (2), Euxyl K712 (3), and Euxyl K903 (4) were added. When all the oil phase ingredients (6-9) were melted, the water phase was added slowly and homogenized. Then the emulsion was cooled below 40°C and ingredients (11,12) and fragrance (13) were added, and the emulsion was homogenized. Afterwards, citric acid (14) was introduced until a pH between 5.0-5.5 was reached and the formulation was homogenized until the proper viscosity was fully developed.

| *Table S3. Chemical compositions of the base face creams.*   \| No \| Trade name \| INCI \| Company \| Cream A  Quantity  (% w/w, ±0.01) \| Cream B  Quantity  (% w/w, ±0.01) \| \| --- \| --- \| --- \| --- \| --- \| --- \| \| 1 \| Water \| *Aqua* \| – \| 73.70 \| 79.70 \| \| 2 \| Sodium gluconate \| *Sodium Gluconate* \| Brenntag \| 0.20 \| 0.20 \| \| 3 \| Euxyl K712 \| *Sodium Benzoate, Potassium Sorbate, Aqua* \| Barentz \| 0.70 \| 0.70 \| \| 4 \| Euxyl K903 \| *Benzyl Alcohol, Benzoic Acid, Dehydroacetic Acid, Tocopherol* \| Barentz \| 0.50 \| 0.50 \| \| 5 \| Olivem 1000 \| *Cetearyl Olivate, Sorbitan Olivate* \| HSH \| 4.00 \| *-* \| \| 6 \| Tegin M Pellets \| *Glyceryl Stearate* \| Adara \| 1.00 \| 1.00 \| \| 7 \| Cetyl alcohol \| *Cetyl alcohol* \| Zarzeccy \| 2.00 \| 2.00 \| \| 8 \| Shea butter \| *Butyrospermum Parkii Butter* \| Zarzeccy \| 2.00 \| 2.00 \| \| 9 \| Grape seed oil \| *Vitis Vinifera Seed Oil* \| Aurum Chemicals \| 6.00 \| 6.00 \| \| 10 \| Jolee 7750 \| *Isoamyl Laurate* \| Surfachem \| 4.00 \| 4.00 \| \| 11 \| Panthenol 75 \| *Panthenol* \| Enzym \| 3.00 \| 3.00 \| \| 12 \| Hialuron DHA/BA 3 \| *Aqua, Sodium Hyaluronate, Hyaluronic Acid, Dehydroacetic Acid, Benzyl Alcohol* \| Mabelle \| 0.50 \| 0.50 \| \| 13 \| Fragrance \| *Parfum* \| Arcon \| 0.20 \| 0.20 \| \| 14 \| Citric acid \| *Citric Acid* \| Brenntag \| 0.20 \| 0.20 \| |
| --- | --- | --- | --- | --- | --- | --- | --- | --- | --- | --- | --- | --- | --- | --- | --- | --- | --- | --- | --- | --- | --- | --- | --- | --- | --- | --- | --- | --- | --- | --- | --- | --- | --- | --- | --- | --- | --- | --- | --- | --- | --- | --- | --- | --- | --- | --- | --- | --- | --- | --- | --- | --- | --- | --- | --- | --- | --- | --- | --- | --- | --- | --- | --- | --- | --- | --- | --- | --- | --- | --- | --- | --- | --- | --- | --- | --- | --- | --- | --- | --- | --- | --- | --- | --- | --- | --- | --- | --- | --- | --- |
